# The biological role of the CXCL12/CXCR4 axis in esophageal squamous cell carcinoma

**DOI:** 10.20892/j.issn.2095-3941.2020.0140

**Published:** 2021-06-15

**Authors:** Xianxian Wu, Hongdian Zhang, Zhilin Sui, Yang Wang, Zhentao Yu

**Affiliations:** 1 Departments of Esophageal Cancer, Tianjin Medical University Cancer Institute and Hospital, National Clinical Research Center for Cancer, Key Laboratory of Cancer Prevention and Therapy, Tianjin, Tianjin’s Clinical Research Center for Cancer, Tianjin 300060, China; 2 Department of Immunology, Tianjin Medical University Cancer Institute and Hospital, National Clinical Research Center for Cancer, Key Laboratory of Cancer Prevention and Therapy, Tianjin, Tianjin’s Clinical Research Center for Cancer, Tianjin 300060, China

**Keywords:** Esophageal squamous cell carcinoma, C-X-C motif chemokine ligand 12, CXC chemokine receptor 4, antagonists, imaging agent

## Abstract

Esophageal cancer is the eighth most common malignant tumor and the sixth leading cause of cancer-related death worldwide. Esophageal squamous cell carcinoma (ESCC) is the main histological type of esophageal cancer, and accounts for 90% of all cancer cases. Despite the progress made in surgery, chemotherapy, and radiotherapy, the mortality rate from esophageal cancer remains high, and the overall 5-year survival rate is less than 20%, even in developed countries. The C-X-C motif chemokine ligand 12 (CXCL12) is a member of the CXC chemokine subgroup, which is widely expressed in a variety of tissues and cells. CXCL12 participates in the regulation of many physiological and pathological processes by binding to its specific receptor, C-X-C motif chemokine receptor type 4 (CXCR4), where it causes embryonic development, immune response, and angiogenesis. In addition, increasing evidence indicates that the CXCL12/CXCR4 axis plays an important role in the biological processes of tumor cells. Studies have shown that CXCL12 and its receptor, CXCR4, are highly expressed in ESCC. This abnormal expression contributes to tumor proliferation, lymph node and distant metastases, and worsening prognosis. At present, antagonists and imaging agents against CXCL12 or CXCR4 have been developed to interfere with the malignant process and monitor metastasis of tumors. This article summarizes the structure, function, and regulatory mechanism of CXCL12/CXCR4 and its role in the malignancy of ESCC. Current results from preclinical research targeting CXCL12/CXCR4 are also summarized to provide a reference for the clinical diagnosis and treatment of ESCC.

## Introduction

Esophageal cancer is the eighth most common cancer worldwide^1,2^, and includes the following two main tissue subtypes: esophageal squamous cell carcinoma (ESCC) and esophageal adenocarcinoma. ESCC, which is the main histological type of esophageal cancer, accounts for approximately 90% of cases worldwide^1^. In recent years, the mortality rate from esophageal cancer has remained high despite advances in surgical techniques, chemotherapy, and radiotherapy strategies. Most patients are diagnosed at an advanced stage, often with lymph node or distant metastasis, and the 5-year survival rate is less than 20%, even in developed countries^3–5^. However, the 5-year survival rate in patients with early diagnosis can be considerably improved using endoscopic or surgical treatment^6^. Therefore, identifying a biomarker that can predict tumorigenesis at an early stage would be very advantageous.

The human chemokine system contains approximately 50 different chemokines and 20 chemokine receptors^7^. Chemokines are soluble small molecule secretory proteins with a molecular weight of approximately 8 kDa. According to the position of their cysteine residues, chemokines can be divided into 4 subgroups (CX3C, CXC, CC, and C, the C represents a cysteine residue, and the X/X3 represents 1 or 3 non-cysteine amino acids at conserved locations)^8,9^. Chemokine receptors are a class of 7 transmembrane spanning domains, G-protein coupled receptors (GPCR), characterized according to their preference for specific chemokines^10,11^. The C-X-C motif chemokine ligand 12 (CXCL12) is one of the most studied CXC chemokine ligands. CXCL12 participates in many aspects of tumor progression, including survival, proliferation, angiogenesis, and metastasis, by interacting with its CXCR4 receptor^12,13^.

The molecular mechanisms involved in the origin and development of ESCC is unclear, and limit the effective treatment options available for this highly invasive tumor. However, in recent years, the role of the CXCL12/CXCR4 axis in ESCC has received increasing attention. Understanding such mechanisms will help to design new targeted therapies to block chemokine-induced metastasis and diffusion. This manuscript aims to describe the role of the CXCL12/CXCR4 axis in promoting the malignant processes in ESCC, and to describe antagonists and imaging agents available for targeting CXCL12/CXCR4, to help guide clinical diagnosis and treatment.

## CXCL12/CXCR4 axis structure and function

The CXCL12 gene is located on chromosome 10q11 and has 6 different splice variants in humans, including α, β, γ, δ, ɛ, and ψ^14^. CXCL12 is a type of homeostatic chemokine, which was originally identified as a pre-B cell growth factor and was found to be essential for homeostatic maintenance^15,16^. CXCL12 is constitutively expressed by bone marrow stromal cells and is therefore referred to as stromal cell-derived factor-1^17^. CXCR4 is a member of the GPCR family, comprising 352 amino acid residues, and is expressed in hematopoietic stem/progenitor, pre-B, and endothelial cells^18–21^. CXCR4 is reported to be upregulated in at least 23 different hematopoietic and nonhematopoietic tumors, including esophageal cancer^22–37^.

The CXCL12/CXCR4 axis is involved in embryonic development, immune and inflammatory responses, and stem cell migration and homing^38–43^. This axis is also involved in the malignant development of various tumors, by promoting proliferation, angiogenesis, invasion, and metastasis^44^. CXCL12 works synergistically with vascular endothelial growth factor to induce neovascularization by attracting endothelial progenitor cells into the tumor microenvironment, resulting in a sufficient oxygen supply for tumor maintenance^25^. CXCL12 can promote the tumor process in four ways: it can promote neovascularization and provide oxygen supply to tumor cells^45^, it can directly promote tumor cell survival and proliferation in a paracrine manner^46^, it can cause tumor cell metastasis by interacting with its receptor (CXCR4), and finally, CXCR4 positive tumor cells may have stem cell characteristics and therefore a high potential for metastasis^47,48^.

## Regulation of the CXCL12/CXCR4 axis in cancer

The expression of CXCL12/CXCR4 can be regulated at three levels: epigenetic, transcriptional, and post-transcriptional. Epigenetic silencing leads to an imbalance in CXCL12/CXCR4 expression, and hypermethylation of the CXCL12 promoter, which is related to its metastatic potential, and has been detected in various tumors^49–51^. However, loss of methylation from the CXCR4 promoter leads to upregulation of CXCR4 expression^52,53^. Tumor cells that maintain CXCR4 overexpression, but lack CXCL12 expression, can be directionally transferred to target organs with high levels of CXCL12 secretion.

Some studies have also found that CXCL12/CXCR4 is regulated by many factors at the transcriptional level. The CXCR4 gene promoter contains the hypoxia response element, and under hypoxic conditions, hypoxia-inducible factor-1 binds to the hypoxia response element region of the promoter, causing the induction of CXCR4 gene transcription and expression^54^. Furthermore, recent studies have reported that nuclear factor kappa-B promotes tumor growth by increasing the expression of CXCL12^55^. Matrix metalloproteinase 10 promotes angiogenesis, growth, and diffusion of human hepatocellular carcinomas by regulating the CXCL12/CXCR4 axis, and the phosphatase and tensin homolog can negatively regulate the expression of CXCL12/CXCR4 in prostate tumors, thereby controlling tumor growth^56–58^. In Jurkat T cells, the lipid phosphatase activity of the phosphatase and tensin homolog negatively regulates CXCR4-mediated chemotaxis. Other factors, such as transforming growth factor β^59^, transcription factor 12^60^, and vascular endothelial growth factor^61^ have all been reported to control the expression of CXCR4, thereby regulating malignant tumor progression.

In addition to the two regulatory mechanisms previously mentioned, CXCL12 and CXCR4 can be regulated by microRNAs (miRNA) at the post-transcriptional level. MiRNAs are a group of highly conserved, small molecular weight noncoding RNAs that participate in the post-transcriptional regulation of gene expression by base complementarity at the 3′-UTRs of target mRNAs, leading to mRNA silencing or transcriptional inhibition^62^. Control of the expression of CXCL12 in cancer cells is due to it being targeted by numerous microRNAs, and this targeting of the CXCL12/CXCR4 axis may inhibit the development and progression of tumors^63–67^.

## The CXCL12/CXCR4 signaling pathway

**Figure 1:**
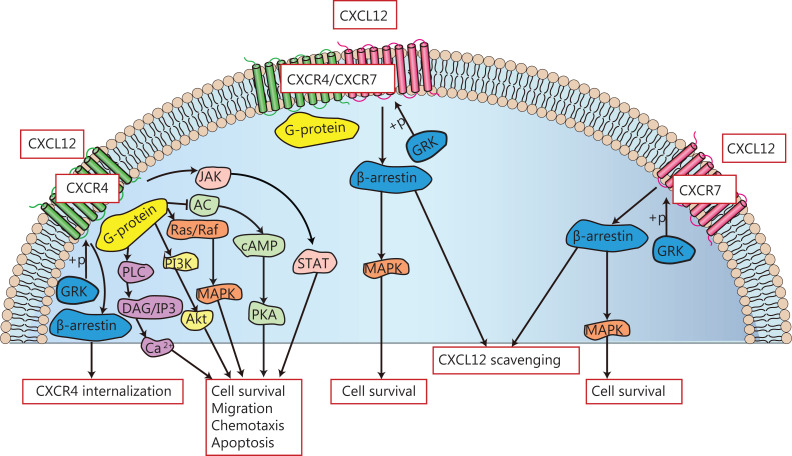
Schematic diagram of the CXCR12/CXCR4 signaling pathway.

The interaction of CXCL12 with its G-protein coupled CXCR4 receptor causes dissociation of its heterotrimeric G protein into Gαi and Gβγ subunits and converts guanosine diphosphate to guanosine triphosphate^68^. Gαi inhibits cAMP production, and causes the inhibition of adenylate cyclase activation, resulting in the activation of downstream pathways, such as phosphatidylinositol 3-kinase/protein kinase B (PI3K/AKT) and mitogen-activated protein kinase (MAPK)^40,69^. Gβγ activates phospholipase C (PLC), which causes the synthesis of diacylglycerol and inositol 1,4,5-triphosphate (IP3). Next, IP3 binds to specific receptors on the endoplasmic reticulum, causing the mobilization of Ca^2+^ from intracellular stores, resulting in a transient increase in intracellular Ca^2+^^70^. This interaction induces the activation of various intracellular pathways, including PI3K-Akt, Ca^2+^-dependent tyrosine kinases such as PYK2, and MAPK signaling pathways, such as P38, JNK, and ERK^71–73^. In addition, related studies have found that CXCL12/CXCR4 can also induce the Janus kinase signal and transcriptional activator (JAK/STAT) pathway independently of G-protein involvement^74^. β-arrestin can also regulate the CXCR4 signaling pathway and mediate CXCR4 receptor internalization and desensitization^75,76^. CXCL12 can also mediate signal transduction through a different receptor, CXCR7. No classical GPCR-mediated signal transduction was observed when CXCR7 was activated by CXCL12; however, the β-arrestin pathway was activated and scavenging of CXCL12 was promoted^77^. CXCR7 can also improve cell survival through the PLC/MAPK signaling pathway^78^. In addition, CXCR7 can change the conformation of the CXCR4/G-protein complex, to form heterodimers with CXCR4, and abrogate the CXCR4-mediated signal transduction^79,80^. The CXCL12-mediated signaling pathway is shown in **Figure 1**.

Studies have also found that expression of CXCR4 in ESCC is higher than that in its corresponding normal tissues. CXCR4 also promotes tumor growth and invasion by activating the PI3K/AKT pathway and upregulating Rho family members^81^. However, the role of the CXCL12/CXCR7 signaling axis in ESCC is unclear.

## The relationship between CXCR4 and ESCC

### CXCR4 expression in ESCC

CXCL12 and CXCR4 are expressed in ESCC, and the level of CXCR4 in primary and metastatic lesions is significantly increased compared to normal tissues. The increased expression levels of CXCL12 and CXCR4 detected by immunohistochemistry in cancer tissues were 50%–78% and 61%–80%, respectively, showing significantly higher levels than found in the corresponding normal tissues^82,83^. Meta-analyses have found that CXCR4 was significantly expressed in ESCC when compared to normal tissues^84^. CXCR4 is a 7 transmembrane spanning domain receptor, and studies have found that it is mainly expressed in the cytoplasm and nucleus^22^. This phenomenon may be due to the activation of CXCR4 by CXCL12 and its translocation from the cell membrane to the cytoplasm, thereby inducing CXCR4 internalization and specific downstream signals. The expression profiles of CXCL12 and CXCR4 are closely related to the biological behavior of ESCC. The expression of CXCL12 affects multiple phenotypes in ESCC, including tumor stage, lymphatic invasion, and distant metastases^83^. The expression of CXCR4 in primary tumors is positively correlated with micrometastases to the lymph nodes and bone marrow, and negatively correlated with overall disease-specific survival^85,86^. Lukaszewicz-Zajac et al.^87^ showed that the concentrations of serum CXCL12 and CXCR4 in patients with esophageal cancers were significantly higher for CXCL12, but significantly lower for CXCR4, when compared to healthy controls. This increase in serum CXCL12 concentration and the concomitant decrease in its receptor may lead to enhancement of the binding capability of CXCR4 for CXCL12.

### CXCR4 promotes ESCC cell metastasis

ESCC is a highly invasive and metastatic tumor with a high tendency for lymph node metastasis. Metastasis of ESCC may involve the CXCR4 receptor because of its unique metastatic pattern and the high constitutive expression of CXCL12 at these metastatic sites^88,89^. Studies have found that CXCR4 is a major chemotactic receptor that mediates lymph node and distant organ metastases in ESCC^90^. The inhibition of CXCR4 with shRNA can significantly reduce the metastatic and proliferative potentials of esophageal cancer^91^.

CXCR4 positive tumor cells metastasize to organs with high expression of CXCL12, such as lymph nodes, liver, lungs, and bone marrow^88,89^. Clinical data from ESCCs have found that the expressions of CXCL12 and CXCR4 are closely related to increased lymph node metastasis, and further studies have confirmed that CXCL12/CXCR4 promotes lymph node metastasis^86^. CXCL12 is highly expressed in lymph nodes and can stimulate lymphangiogenesis by inducing the migration of lymphangiogenic endothelial cells. The expression of CXCR4 is upregulated in lymphangiogenic endothelial cells, but the expression level is low in mature lymphatic vessels^92^.

### The role of CXCR4 in ESCC cell proliferation

In addition to metastasis, the CXCL12/CXCR4 signaling pathway also contributes to the growth and proliferation of ESCC^93^. When the CXCR4 knockdown cell line, Eca109, was inoculated subcutaneously into BALB/c-nu/nu mice, the tumor mass formed at the inoculation site was significantly smaller than the control group. Moreover, *in vitro* experiments showed that the proliferative capability of Eca109-shCXCR4 cells was significantly lower than that of the parent or control cells. The proliferative capability of human esophageal epithelial cells overexpressing CXCR4 was increased when compared to the control group^81^.

The molecular mechanisms by which CXCR4 promotes tumor growth have been widely studied and are summarized as follows: (1) CXCL12 produced in the tumor microenvironment allows the entrance of circulating endothelial progenitor cells to the primary tumor site and produces additional microvessels, thereby increasing the oxygen supply to the tumor^14,94^. (2) Common signaling pathways related to cell proliferation, such as PI3K/AKT, Src/ERK1-2, and STAT3, are activated^81,95,96^. (3) CXCR4/CXCL12 signaling participates in Treg cell bone marrow homing and plasmacytoid dendritic cell trafficking, suppressing antitumor immunity, and promoting tumor growth^97–99^.

### CXCR4 and ESCC prognosis

Studies have evaluated the effect of CXCR4 expression on ESCC prognosis. Gockel et al.^100^ found that the median survival time for patients with high expression of CXCR4 was 20 months, whereas patients with low expression was 76 months, and this difference was statistically significant (*P* < 0.05). Furthermore, Wu et al.^101^ conducted a meta-analysis comprising 1,055 participants from Germany, China, and Japan. Their results showed that overexpression of CXCR4 was related to tumor depth (*P* < 0.01), lymph node status (*P* < 0.01), tumor node metastasis stage (*P* < 0.01), and histological type (*P* = 0.03). They also found that the overexpression of CXCR4 significantly decreased the overall survival rate (*P* < 0.01). CXCR4 therefore appears to be a reliable prognostic marker for ESCC.

*In vivo* and *in vitro* studies have shown that lentiviral shRNA-induced CXCR4 gene silencing can inhibit the proliferation and metastasis of ESCC cell lines^91^. However, the specific mechanism by which CXCR4 promotes the malignant development of ESCC is unclear. Recent studies have found a rare population of tumor cells whose characteristics are like stem cells. These cells have been referred to as cancer stem cells (CSCs), which mediate tumor growth, metastasis, recurrence, and therapeutic drug resistance. CXCR4 is highly expressed in esophageal tumor stem cells, and the expression of CXCR4 in CSCs increases the malignant potential of ESCC and is associated with ESCC recurrence and metastasis^102^. CD133+ esophageal CSCs contain a subgroup of cells characterized by the co-expression of CXCR4 and can evade the primary tumor and establish distant metastasis^47^.

### Future therapeutic directions

The overexpression of CXCR4 in tumor tissue is related to tumor proliferation, tumor invasion, increased risk of metastasis, and poor survival outcomes. Many small molecule CXCR4-based inhibitors have been developed because of the important role of CXCR4 in oncology. These inhibitors have been reviewed in several studies. CXCR4 antagonists can be divided into the following 4 categories: small peptide CXCR4 antagonists, non-peptide CXCR4 antagonists, antibodies against CXCR4, and SDF-1-modified agonists and antagonists.

AMD3100 (Mozobil™) was originally designed for the treatment of AIDS^103,104^. However, an increase in leukocyte count was observed in phase I clinical trials after AMD3100 administration. Further studies found that AMD3100 could mobilize CD34+ hematopoietic stem cells in peripheral blood and when combined with granulocyte colony-stimulating factor, could recruit more CD34+ cells than granulocyte colony-stimulating factor alone^105,106^. The US Food and Drug Administration (FDA) has currently approved AMD3100 for the treatment of patients with hematological malignancies^106^.

CTCE-9908 is a small peptide analog designed to antagonize the CXCR4 receptor and can reduce the lung metastasis of osteosarcoma and melanoma cells in a mouse model^107^. CTCE-9908 has been tested in phase I/II clinical trials as monotherapy for solid tumors. In July 2005, the FDA approved CTCE-9908 as an orphan drug for the treatment of osteosarcoma.

Olaptesed Pegol (NOX-A12) is an L-stereoisomer RNA oligonucleotide, linked to 40 kDa polyethylene glycol, and this anti-CXCL12 compound can bind and neutralize CXCL12 with high affinity and specificity. NOX-A12 mobilizes hematopoietic stem cells and leukocytes from the bone marrow to the periphery^108^. In 2014, NOX-A12 combined with radiotherapy was approved by the FDA for the treatment of malignant glioma and is currently being tested in phase IIa clinical trials for the treatment of multiple myeloma and chronic lymphoblastic leukemia^109,110^. In addition, other CXCR4 inhibitors are in different stages of clinical trials (**Table 1**).

**Table 1:** Clinical trials using CXCL12/CXCR4 pathway inhibitors

Description	Drug name	Indications	Study phase	Clinical trials No.
Non-peptide small molecules CXCR4 antagonists	AMD3100 (plerixafor)	FDA-approved for HSC mobilization in NHL and MM		
		Glioblastoma	1	NCT01339039
		Ewing sarcoma, neuroblastoma, brain tumours	1,2	NCT01288573
	MSX-122	Refractory metastatic or locally advanced solid tumor	1	NCT00591682
	TG-0054 (burixafor)	MM/NHL/HD HSC	2	NCT01018979
		MM/NHL/HD	2	NCT01458288
Peptide CXCR4 antagonists	BKT-140	Multiple myeloma stem cell mobilization	1,2	NCT01010880
CXCL12-based peptides	CTCE-9908	FDA-assigned for the treatment of osteosarcoma		
CXCL12-targeted agents	NOX-A12	Dosage test healthy patient for HSCT (+/– filgrastim)	1	NCT00976378NCT01194934
Antibodies to CXCR4	Ulocuplumab (BMS936564/MDX-1338)	Acute myeloid leukemia	1	NCT01120457NCT0135965
Nanobodies	ALX-0651	HSC mobilization, HIV	1	NCT01374503

HCS, hematopoietic stem cells; NHL, non-Hodgkin lymphoma; MM, multiple myeloma; HD, Hodgkin disease; HSCT, HSC transplantation.

CXCR4 antagonists play an important role in sensitizing tumor cells to chemotherapy, and existing imaging agents targeting CXCR4 have the potential to guide and monitor cancer treatment. The overexpression of CXCR4 in cancer directly affects the chemotaxis of tumor cells to the SDF-1 gradient. Highly expressed CXCL12 has been found in the most common sites of tumor metastasis, including lymph nodes, lung, liver, and bone marrow. Thus, the application of CXCR4 in the field of diagnostic oncology has received increasing attention. Nimmagadda et al.^111^ used Iodine-125-labeled anti-CXCR4 monoclonal antibody to develop CXCR4-targeted tracers. Hanaoka et al.^112^ revealed that the peptide antagonist, Ac-TZ14011, labeled with Indium-111 is a potential agent for imaging the expression levels of CXCR4 in metastatic tumors *in vivo*. In addition, AMD3100 has been labeled with copper 64 to visualize CXCR4-positive tumor cells *in vivo* and can be used to guide and monitor anti-CXCR4 tumor treatment^113^. Several other agents, such as CXCL12-based imaging agent and bioluminescence, have been developed for tumor diagnostic imaging^114–116^.

For locally advanced and unresectable ESCC patients, the combination therapy of fluoropyrimidine-based and platinum-based drugs is the first-line treatment. Persistent high expression of CXCR4 after chemoradiotherapy predicts tumor recurrence and poor prognosis^117^. Blocking the CXCL12-CXCR4 axis can enhance the sensitivity of tumor cells to chemotherapy drugs and reduce tumor volume^82,93,118,119^. In addition, it has been reported that AMD3100 can regulate immunosuppression in tumors^120,121^. These results indicate that blocking the CXCL12/CXCR4 axis is a potential target to improve the prognosis of ESCC on the basis of existing chemotherapy, radiotherapy, or immunotherapy.

## Conclusions

Overall, current data show that the CXCL12/CXCR4 axis promotes the proliferation, invasion, and metastasis of ESCC, resulting in poor patient prognosis. However, the efficacy of CXCR4 antagonists or imaging agents in ESCC has not been fully tested in clinical trials. Additional research and clinical evaluations are therefore needed to determine the benefits of CXCL12/CXCR4 antagonism and imaging agents in patients with ESCC.
